# ﻿Soil-borne Ophiostomatales species (Sordariomycetes, Ascomycota) in beech, oak, pine, and spruce stands in Poland with descriptions of *Sporothrixroztoczensis* sp. nov., *S.silvicola* sp. nov., and *S.tumida* sp. nov.

**DOI:** 10.3897/mycokeys.97.97416

**Published:** 2023-05-16

**Authors:** Piotr Bilański, Robert Jankowiak, Halvor Solheim, Paweł Fortuna, Łukasz Chyrzyński, Paulina Warzecha, Stephen Joshua Taerum

**Affiliations:** 1 Department of Forest Ecosystems Protection, University of Agriculture in Krakow, Al. 29 Listopada 46, 31-425 Krakow, Poland University of Agriculture in Krakow Krakow Poland; 2 Norwegian Institute of Bioeconomy Research, P.O. Box 115, 1431, Ås, Norway Norwegian Institute of Bioeconomy Research Ås Norway; 3 The Connecticut Agricultural Experiment Station, Department of Plant Pathology and Ecology, Jenkins-Waggoner Laboratory, 123 Huntington Street P.O. Box 1106, New Haven, CT 06504-1106, USA The Connecticut Agricultural Experiment Station, Department of Plant Pathology and Ecology, Jenkins-Waggoner Laboratory New Haven United States of America

**Keywords:** 3 new taxa, ophiostomatalean fungi, phylogenetics, *Pinussylvestris*, soil-inhabiting fungi, *Sporothrix*, taxonomy

## Abstract

Ophiostomatales (Ascomycota) contains many species, most of which are associated with bark beetles. Some members of this order are plant or animal pathogens, while others colonize soil, different plant tissues, or even carpophores of some Basidiomycota. However, little is known about soil-inhabiting Ophiostomatales fungi. A survey of these fungi associated with soil under beech, oak, pine, and spruce stands in Poland yielded 623 isolates, representing 10 species: *Heinzbutiniagrandicarpa*, *Leptographiumprocerum*, *L.radiaticola*, *Ophiostomapiliferum*, *O.quercus*, *Sporothrixbrunneoviolacea*, *S.dentifunda*, *S.eucastaneae*, and two newly described taxa, namely *Sporothrixroztoczensis***sp. nov.** and *S.silvicola***sp. nov.** In addition, isolates collected from fallen shoots of *Pinussylvestris* that were pruned by *Tomicus* sp. are described as *Sporothrixtumida***sp. nov.** The new taxa were morphologically characterized and phylogenetically analyzed based on multi-loci sequence data (ITS, β-tubulin, calmodulin, and translation elongation factor 1-α genes). The Ophiostomatales species were especially abundant in soil under pine and oak stands. *Leptographiumprocerum*, *S.silvicola*, and *S.roztoczensis* were the most frequently isolated species from soil under pine stands, while *S.brunneoviolacea* was the most abundant in soil under oak stands. The results highlight that forest soil in Poland has a wide diversity of Ophiostomatales taxa, but further studies are required to uncover the molecular diversity and phylogenetic relationships of these fungi, as well as their roles in soil fungal communities.

## ﻿Introduction

Ophiostomatales (Sordariomycetidae, Ascomycota) contains a single family, the Ophiostomataceae, which includes 16 well-defined genera together with many taxa of uncertain phylogenetic position. *Leptographium*, *Ophiostoma*, and *Sporothrix* represent the genera with the largest numbers of taxa, which are grouped into species complexes based on morphology and phylogenetic relationships. These fungi are characterized by the presence of globose ascomata with short to very long necks and ascospores that vary in size and shape, mostly allantoid, bacilliform, and cylindrical with sheaths. The asexual morphs exhibit five conidiophore types: hyalorhinocladiella-like, leptographium-like, pesotum-like, raffaelea-like, and sporothrix-like. The species in this order are best known as wood-inhabiting fungi that live in association with various arthropods, but many species can also occupy other habitats such as soil, carpophores, plant infructescences or animal tissues (de Beer and Wingfield 2013; de Beer et al. 2013a, b, 2016, 2022).

Little is known about the diversity of Ophiostomatales species in different soil ecosystems, although some *Sporothrix* spp. have been reported in soil worldwide. The currently known soil-inhabiting species include *S.aurorae* (X.D. Zhou & M.J. Wingf.) Z.W. de Beer, T.A. Duong & M.J. Wingf., *S.bragantina* (Pfenning & Oberw.) Z.W. de Beer, T.A. Duong & M.J. Wingf., *S.brasiliensis* Marimon, Gené, Cano & Guarro, *S.brunneoviolacea* Madrid, Gené, Cano & Guarro, *S.chilensis* A.M. Rodrigues, Choappa, G.F. Fernandes, de Hoog & Z.P. de Camargo, *S.dimorphospora* (Roxon & S.C. Jong) Madrid, Gené, Cano & Guarro, *S.globosa* Marimon, Cano, Gené, Deanna A. Sutton, H. Kawas. & Guarro, *S.guttiliformis* de Hoog, *S.humicola* de Mey., Z.W. de Beer & M.J. Wingf., *S.inflata* de Hoog, ‘*S.inflata* 2’, *S.luriei* (Ajello & Kaplan) Marimon, Gené, Cano & Guarro, *S.mexicana* Marimon, Gené, Cano & Guarro, *S.narcissi* (Limber) Z.W. de Beer, T.A. Duong & M.J. Wingf., *S.pallida* (Tubaki) Matsush., *S.schenckii* Hektoen & C.F. Perkins, *S.stenoceras* (Robak) Z.W. de Beer, T.A. Duong & M.J. Wingf., and *S.stylites* de Mey., Z.W. de Beer & M.J. Wingf. (de Beer et al. 2003, 2016). Among them, *S.brunneoviolacea*, *S.dimorphospora* (Madrid et al. 2010), *S.inflata*, ‘*S.inflata* 2’ (de Hoog 1974; de Beer et al. 2016), *S.pallida*, *S.schenckii* (de Meyer et al. 2008; de Beer et al. 2016), and *S.stenoceras* (Novotný and Šrůtka 2004) have been reported from European soils. Some of these soil-borne species, namely *S.brasiliensis*, *S.chilensis*, *S.globosa*, *S.luriei*, and *S.schenckii*, are agents of human and animal sporotrichosis (Lòpez-Romero et al. 2011; Zhang et al. 2015; Rodrigues et al. 2017; Ramírez-Soto et al. 2018).

Members of the Ophiostomatales are typically tree- or wood-infecting fungi, and are commonly associated with bark- and wood-dwelling beetles and their associated mites (de Beer et al. 2022). The association between these fungi and subcortical insects has been extensively investigated in Poland (e.g. Jankowiak and Kolařík 2010; Jankowiak and Bilański 2013a, b, c; Jankowiak et al. 2017, 2019a). Polish and South African studies have demonstrated that wounded hardwoods provide habitat for a large diversity of Ophiostomatales species (Musvuugwa et al. 2016; Jankowiak et al. 2019b). The findings from studies in Poland also provided the first evidence that European nitidulid beetles act as effective vectors of *Ophiostoma* spp. and *Sporothrix* spp. (Jankowiak et al. 2019b). The Polish surveys led to the discovery and description of many Ophiostomatales species (e.g. Linnakoski et al. 2016; Aas et al. 2018; Jankowiak et al. 2018a, 2019c, 2020, 2021; Strzałka et al. 2020; Ostafińska et al. 2021).

Previous studies of soil-borne fungi belonging to the Ophiostomatales were limited to *Sporothrix* species (e.g. de Meyer et al. 2008; Madrid et al. 2010) and even this genus remains largely unstudied. The aim of this study was to explore the diversity of Ophiostomatales members associated with soil under forest trees in Poland from a taxonomic perspective and to describe potential resultant new species. Fungi were baited with branch fragments that were buried in soil under beech, oak, pine, and spruce forests. We also describe a *Sporothrix* species that was isolated from fallen shoots of *Pinussylvestris* L. mentioned in a previously published study (Jankowiak and Kolařík 2011).

## ﻿Materials and methods

### ﻿Study area

Wood samples were collected from four forest districts located in southern Poland (Józefów, Krzeszowice, Siewierz, and Węgierska Górka) between 2015–2019. In each district, 10 stands dominated by *Fagussylvatica* L. (Krzeszowice, Małopolskie Province), *Piceaabies* (L.) H. Karst. (Węgierska Górka, Śląskie Province), *P.sylvestris* (Józefów, Lubelskie Province), and *Quercusrobur* L. (Siewierz, Śląskie Province) were selected, making a total of 40 stands (10 stands for each tree species). The stands were managed and between 35 to 135 years of age.

All sampled stands have temperate climates. Węgierska Górka is located in the lower montane belt of the Western Carpathians (607–896 m a.s.l.) with an average annual temperature and precipitation of 6.5 °C and approximately 950 mm, respectively. The other forest stands are in the Highlands of Poland (217–347 m a.s.l.) with average annual temperature and precipitation of 7–8 °C and approximately 600–800 mm, respectively. Tree-stratum vegetation in Józefów is dominated by *P.sylvestris*, but also consists of *Abiesalba* Mill., *Alnusglutinosa* (L.) Gaertn., *Betulapendula* Roth, *P.abies*, and *Q.robur*. In Krzeszowice, *F.sylvatica* is the dominant tree species, but other species are also present, such as *Carpinusbetulus* L., *P.sylvestris*, and *Q.robur*. Siewierz stands are dominated by *Q.robur*, but also include *Acerpseudoplatanus* L., *A.glutinosa*, *B.pendula*, *C.betulus*, *Larixdecidua* Mill., *P.abies*, and *P.sylvestris*. Finally, vegetation in Węgierska Górka is dominated by *P.abies*, but also contains *A.alba* and *F.sylvatica*.

Soil samples for laboratory analyses were collected from each stand (10 samples per stand, for a total of 400 samples). The samples were collected from the humus A mineral horizon (10 cm deep) after the upper organic O horizon was removed. Freshly collected soil samples were dried and then sieved through a 2 mm mesh sieve. The particle size distribution was analyzed using a laser diffraction method (Analysette 22, Fritsch, Idar-Oberstein, Germany). The pH of soil samples in H_2_O and KCl was determined by a potentiometric method (Ostrowska et al. 1991). All stands were characterized by high soil acidity, with the pH ranging from 3.81 to 6.10 (in H_2_O) and 2.91 to 5.67 (in KCl). After air-drying, soil samples were sifted through a sieve with a mesh diameter of 2 mm. The particle size distribution was determined using laser diffraction (Analysette 22, Fritsch, Idar-Oberstein, Germany). Soil textures were sandy in 27 stands and silty in the remaining 13 stands.

### ﻿Isolation of fungi

Fungi were isolated using branches (25 cm × 5 cm × 5 cm) of *F.sylvatica*, *P.abies*, *P.sylvestris*, and *Q.robur* that were cut along the axes. Healthy branches were taken from trees that represented the dominant species in each stand; for example, in stands dominated by *F.sylvatica*, only its branches were used. Each branch was autoclaved in a sterile plastic bag and was stored for 1–2 days at a temperature of 5 °C. They were then removed from the bags and immediately placed in the soil. Ten sterilized branches were placed in each stand. Branches were buried in the humus mineral A horizon after the organic O horizon was removed, at random locations in the stands (Suppl. material 1: fig. S1). There is no information about the occurrence of Ophiostomatales species in specific soil levels. We have used the humus mineral horizon (A) because this level is characterized by high thermal and humidity stability (Ekici et al. 2014; Neto et al. 2017). Due to potential fungal infection from roots, the branches were placed 2 m away from tree roots. The branches were buried after the main flight period of the root-feeding bark beetles to avoid colonization by insects carrying other Ophiostomatales species (Jankowiak and Bilański 2013a) and were retrieved two months after they were initially buried. After removal, the branches were placed in separate sterile bags and moved to the laboratory of Robert Jankowiak at the University of Agriculture in Krakow, Poland (Suppl. material 1: fig. S2). A total of 400 samples were collected during the study from every stand type (100 from beech stands, 100 from oak, 100 from pine, and 100 from spruce). No signs of insect presence (adults, larvae, bites, wood holes, galleries) were visible on any branch.

The branches were washed under tap water and dried on blotting paper and covered with cotton wool saturated with 96% ethanol for 15 seconds to sterilize the wooden surfaces. A sterile wood chisel was then used to remove the surface of the wood up to a depth of 2 mm. From each block, six small fragments of discolored wood (4 × 4 mm) were taken with a sterile chisel and placed in Petri dishes containing 2% malt extract agar (MEA; Biocorp Polska Sp. z o.o., Warszawa, 20 g Biocorp malt extract, 20 g Biocorp agar, and 1000 mL sterile water) amended with cycloheximide (200 mg/L, Aldrich-Sigma, St. Louis, Co. LLC.) and tetracycline (50 mg/L, Aldrich-Sigma, St. Louis, Co. LLC). Based on the preliminary morphological investigation, emerging cultures resembling members of the Ophiostomatales were purified by transferring small pieces of mycelium or spore masses from individual colonies to fresh 2% MEA. Cultures were incubated at room temperature in the dark at 22 °C. After two weeks of incubation, the purified fungal cultures were grouped into morphotypes. Depending on the number of isolates that belonged to the same morphotype, 1–12 isolates per morphotype were chosen for molecular identification (Table 1). In the end, the isolates were categorized into ten morphotypes.

**Table 1.** Isolates from this study used in the phylogenetic analyses.

Taxon no.	Fungal species	Isolate no^A^			Source	Site	GenBank accessions^B^				
		CBS	CMW	KFL			ITS	LSU	*TUB*2	*TEF*1	*CAL*
1	*Heinzbutiniagrandicarpa*			KFL23PFDb	Wood buried in soil of *Quercusrobur* stand	Siewierz	OP594819		OP588965	OP589005	
2	*Leptographiumprocerum*			KFL42So	Wood buried in soil of *Pinussylvestris* stand	Józefów			OP588956		
				KFL51So	Wood buried in soil of *Pinussylvestris* stand	Józefów			OP588957		
				KFL59So	Wood buried in soil of *Pinussylvestris* stand	Józefów		OP594816	OP588958	OP589002	
				KFL62So	Wood buried in soil of *Pinussylvestris* stand	Józefów		OP594817	OP588959	OP589003	
				KFL68So	Wood buried in soil of *Pinussylvestris* stand	Józefów		OP594818	OP588960	OP589004	
				KFL70So	Wood buried in soil of *Pinussylvestris* stand	Józefów			OP588961		
				KFL77So	Wood buried in soil of *Pinussylvestris* stand	Józefów			OP588962		
				KFL94So	Wood buried in soil of *Pinussylvestris* stand	Józefów			OP588963		
				KFL104So	Wood buried in soil of *Pinussylvestris* stand	Józefów			OP588964		
3	*Leptographiumradiaticola*			KFL6So	Wood buried in soil of *Pinussylvestris* stand	Józefów		OP594813	OP588952	OP588998	
				KFL15So	Wood buried in soil of *Pinussylvestris* stand	Józefów			OP588953	OP588999	
				KFL16So	Wood buried in soil of *Pinussylvestris* stand	Józefów		OP594814	OP588954	OP589000	
				KFL65So	Wood buried in soil of *Pinussylvestris* stand	Józefów		OP594815	OP588955	OP589001	
4	*Ophiostomapiliferum*			KFL6Sob	Wood buried in soil of *Pinussylvestris* stand	Józefów	OP594820		OP588966	OP589006	
				KFL11So	Wood buried in soil of *Pinussylvestris* stand	Józefów	OP594821		OP588967	OP589007	
5	*Ophiostomaquercus*			KFL5Db	Wood buried in soil of *Quercusrobur* stand	Siewierz	OP594822		OP588968	OP589008	
				KFL10Db	Wood buried in soil of *Quercusrobur* stand	Siewierz	OP594823		OP588969		
				KFL55Db	Wood buried in soil of *Quercusrobur* stand	Siewierz	OP594824		OP588970		
6	*Sporothrixbrunneoviolacea*			KFL64PFDb	Wood buried in soil of *Quercusrobur* stand	Siewierz	OP594825		OP588971	OP589009	OP589035
				KFL16PFDb	Wood buried in soil of *Quercusrobur* stand	Siewierz	OP594826		OP588972	OP589010	OP589036
				KFL32PFaDb	Wood buried in soil of *Quercusrobur* stand	Siewierz	OP594827		OP588973	OP589011	OP589037
				KFL41PFDb	Wood buried in soil of *Quercusrobur* stand	Siewierz	OP594828		OP588974	OP589012	OP589038
				KFL19PFaDb	Wood buried in soil of *Quercusrobur* stand	Siewierz	OP594829		OP588975	OP589013	OP589039
				KFL65PFDb	Wood buried in soil of *Quercusrobur* stand	Siewierz	OP594830		OP588976	OP589014	OP589040
				KFL19PFbDb	Wood buried in soil of *Quercusrobur* stand	Siewierz	OP594831		OP588977	OP589015	OP589041
				KFL20PFDb	Wood buried in soil of *Quercusrobur* stand	Siewierz	OP594832		OP588978	OP589016	OP589042
				KFL89PFDb	Wood buried in soil of *Quercusrobur* stand	Siewierz	OP594833		OP588979	OP589017	OP589043
7	*Sporothrixdentifunda*			KFL21PFaDb	Wood buried in soil of *Quercusrobur* stand	Siewierz	OP594834		OP588980	OP589018	OP589044
				KFL21PFbDb	Wood buried in soil of *Quercusrobur* stand	Siewierz	OP594835		OP588981		
				KFL28PFDb	Wood buried in soil of *Quercusrobur* stand	Siewierz	OP594836		OP588982	OP589019	OP589045
				KFL37PFDb	Wood buried in soil of *Quercusrobur* stand	Siewierz	OP594837		OP588983	OP589020	
8	*Sporothrixeucastaneae*			KFL54PFDb	Wood buried in soil of *Quercusrobur* stand	Siewierz	OP594838		OP588984	OP589021	OP589046
9	*Sporothrixroztoczensis* sp. nov.			KFL36So	Wood buried in soil of *Pinussylvestris* stand	Józefów	OP594846		OP588992	OP589029	OP589054
		147973	57307	KFL96So^T^	Wood buried in soil of *Pinussylvestris* stand	Józefów	OP594847	OQ449632	OP588993	OP589030	OP589055
		147972	57306	KFL78So^C^	Wood buried in soil of *Pinussylvestris* stand	Józefów	OP594848	OQ449633	OP588994	OP589031	OP589056
		147974	57308	KFL89So	Wood buried in soil of *Pinussylvestris* stand	Józefów	OP594849		OP588995	OP589032	OP589057
10	*Sporothrixsilvicola* sp. nov.			KFL85PFDb	Wood buried in soil of *Quercusrobur* stand	Siewierz	OP594839		OP588985	OP589022	OP589047
				KFL3So	Wood buried in soil of *Pinussylvestris* stand	Józefów	OP594840		OP588986	OP589023	OP589048
		149238		KFL5So	Wood buried in soil of *Pinussylvestris* stand	Józefów	OP594841		OP588987	OP589024	OP589049
		149241		KFL48So^T^	Wood buried in soil of *Pinussylvestris* stand	Józefów	OP594842	OQ449630	OP588988	OP589025	OP589050
		149239		KFL38So	Wood buried in soil of *Pinussylvestris* stand	Józefów	OP594843		OP588989	OP589026	OP589051
		149240		KFL116So^C^	Wood buried in soil of *Pinussylvestris* stand	Józefów	OP594844	OQ449631	OP588990	OP589027	OP589052
		149242		KFL36Sw	Wood buried in soil of *Piceaabies* stand	Andrychów	OP594845		OP588991	OP589028	OP589053
11	*Sporothrixtumida* sp. nov.	147970	57304	KFL55RJ^TD^	Shoots of Scots pine pruned by *Tomicus* sp.	Mielec	OP594850	OQ449634	OP588996	OP589033	OP589058
		147971	57305	KFL85RJ^CD^	Shoots of Scots pine pruned by *Tomicus* sp.	Mielec	OP594851	OQ449635	OP588997	OP589034	OP589059

^A^CBS = Westerdijk Fungal Biodiversity Institute, Utrecht, The Netherlands; CMW = Culture Collection of the Forestry and Agricultural Biotechnology Institute (FABI), University of Pretoria, Pretoria, South Africa; KFL = Culture collection of the Department of Forest Ecosystems Protection; University of Agriculture in Krakow, Poland. ^B^ITS = internal transcribed spacer region of the nuclear ribosomal DNA gene; LSU = internal transcribed spacer region 2 and the 28S large subunit of the nrDNA gene; *TUB*2 = β-tubulin; *TEF*1 = Translation elongation factor 1-alpha; CAL = calmodulin. ^C^ additional specimen examined. ^D^ Isolates collected during previous surveys in Poland and identified as *Sporothrix* sp. 1 (Jankowiak and Kolařík 2011). ^T^ denotes ex-type cultures.

The collection details for the *Sporothrix* species isolated from fallen shoots of *P.sylvestris* (Table 1) are described in a study by Jankowiak and Kolařík (2011). The cultures are maintained in the culture collection of the Department of Forest Ecosystems Protection, University of Agriculture in Krakow, Poland. The ex-type isolates and representative isolates of the new species described were deposited in the culture collection (**CBS**) of the Westerdijk Fungal Biodiversity Institute, Utrecht, The Netherlands and in the culture collection (**CMW**) of the Forestry and Agricultural Biotechnology Institute (**FABI**) at the University of Pretoria, South Africa. Dried cultures were deposited as holotype specimens in the Mycological Herbarium (**O**) of the Natural History Museum at the University of Oslo, Norway.

### ﻿Microscopy and growth studies

Morphological characters were examined for selected isolates including the type specimens. Cultures were grown on 2% Malt Extract Agar (MEA) made up of 20 g Bacto malt extract and 20 g Bacto agar powder (Becton Dickinson and Company, Franklin Lakes, USA) in 1 L of deionized water. In attempts to induce ascomata formation, autoclaved twigs of host trees were placed at the centres of agar plates containing 2% MEA. To promote the production of ascomata, single conidial isolates were crossed following the technique described by Grobbelaar et al. (2009). These cultures were incubated at 25 °C and monitored regularly for the appearance of developing structures.

Samples of fungal tissues were placed in 80% lactic acid on glass slides, and developing structures were observed using a Nikon Eclipse 50*i* microscope (Nikon Corporation, Tokyo, Japan) with an Invenio 5S digital camera (DeltaPix, Maalov, Denmark) to capture photographic images. Color designations were based on the color charts of Kornerup and Wanscher (1978). For each taxonomically relevant structure, fifty measurements were made, when possible, using the Coolview 1.6.0 software (Precoptic, Warsaw, Poland). Averages, ranges, and standard deviations were presented in the format ‘(min–)(mean–SD)–(mean+SD)(–max)’.

Growth characteristics of the novel species were determined by analyzing the radial growth for two isolates per species. Agar disks (5 mm in diameter) were cut from the actively growing margins of fungal colonies and these disks were placed at the centres of plates containing 2% MEA. Four replicate plates for each isolate of the three putative new species were incubated in the dark. The isolates were grown at 5, 10, 15, 20, 25, 30 and 35 °C. The radial growth was determined 14 days after inoculation, and growth rates were calculated as mm/day.

### ﻿PCR, sequencing, and phylogenetic analyses

DNA was extracted using the Genomic Mini AX Plant Kit (A&A Biotechnology, Gdynia, Poland) according to the manufacturer’s protocol. For fungi that resided in the genus *Leptographium*, the nuclear large subunit (LSU) region was amplified using the primers LR0R and LR5 (Vilgalys and Hester 1990), the β-tubulin (*TUB*2) gene was amplified using the primers Bt2a and Bt2b (Glass and Donaldson 1995), and the elongation factor 1-α (*TEF*1) gene was amplified using the primers EF2F (Marincowitz et al. 2015) and EF2R (Jacobs et al. 2004). For all other fungi, the internal transcribed spacer regions 1 and 2 (ITS), including the 5.8S region, were amplified using the primers ITS1F and ITS4 (White et al. 1990; Gardes and Bruns 1993), the *TUB*2 gene was amplified using the primers Bt2a and Bt2b (Glass and Donaldson 1995), and the *TEF*1 gene was amplified using the primers F-728F (Carbone and Kohn 1999) and EF2 (O’Donnell et al. 1998). In addition, the calmodulin (CAL) gene was amplified with the primer pairs CL1 and CL2a (O’Donnell et al. 2000) or CL3F and CL3R (de Beer et al. 2016) for fungi that reside in the genus *Sporothrix*. For new *Sporothrix* species, LSU region was amplified using the primers LR0R and LR5 (Vilgalys and Hester 1990). PCR and sequencing were conducted following the protocols described by Jankowiak et al. (2019c). All sequences obtained in this study were deposited in GenBank. The obtained ITS/LSU sequences were compared with sequences in NCBI GenBank for preliminary identifications and were used to determine generic placement in the Ophiostomatales. For *Leptographium* and *Ophiostoma* spp. the *TUB*2 and *TEF*1 datasets were analyzed separately for each species complex. For *Sporothrix* spp., the *CAL*, *TUB*2 and *TEF*1 datasets were analyzed across the entire genus.

Phylogenetic trees were generated independently for each gene. Resulting trees were visually compared for topological incongruences. Genes showing no topological incongruence for *Sporothrix* spp. were combined and analyzed as a concatenated dataset. Sequence alignments were performed using the online version of MAFFT v7 (Katoh and Standley 2013). The ITS, LSU, *TUB*2, *CAL*, and *TEF*1 datasets were aligned using the E-INS-i strategy with a 200PAM/k=2 scoring matrix, a gap opening penalty of 1.53 and an offset value of 0.00. The alignments were checked manually with BioEdit v.2.7.5 (Hall 1999). Phylogenetic trees were inferred for each of the datasets using three different methods: Maximum likelihood (ML), Maximum Parsimony (MP), and Bayesian inference (BI). For ML and BI analyses, the best-fit substitution models for each aligned dataset were established using the corrected Akaike Information Criterion (AIC) in jModelTest 2.1.10 (Guindon and Gascuel 2003; Darriba et al. 2012). ML analyses were carried out with PhyML 3.0 (Guindon et al. 2010), utilizing the Montpelier online server (http://www.atgc-montpellier.fr/phyml/). Node support values and the overall reliability of the ML tree topology were assessed using 1000 bootstrap pseudoreplicates.

MP analyses were performed using PAUP* 4.0b10 (Swofford 2003). Gaps were treated as a fifth state. Confidence levels for the nodes within the inferred tree topologies were determined using 1000 bootstrap replicates. Tree bisection and reconnection (TBR) were selected as the branch swapping option. The tree length (TL), Consistency Index (CI), Retention Index (RI), Homoplasy Index (HI), and Rescaled Consistency Index (RC) were recorded for each analyzed dataset after the trees were generated.

BI analyses using Markov Chain Monte Carlo (MCMC) methods were carried out with MrBayes v3.1.2 (Ronquist and Huelsenbeck 2003). Four MCMC chains were run for 10 million generations applying the best-fit model for each dataset. Trees were sampled every 100 generations, resulting in 100,000 trees. Tracer v1.4.1 (Rambaut and Drummond 2007) was used to determine the burn-in value for each dataset. The remaining trees were used to generate a 50% majority rule consensus tree, which allowed for calculating posterior probability values for the nodes. All alignments and trees were deposited into TreeBASE (Reviewer access URL: http://purl.org/phylo/treebase/phylows/study/TB2:S29855?x-access-code=62dd9f4ad30f131a52104b44860daf9e&format=html).

## ﻿Results

### ﻿Collections of fungi

In total, 623 Ophiostomatales isolates were obtained from 2400 wooden samples (six pieces from each of 400 branches; Table 2). Five hundred and forty-one isolates were collected from pine, 79 isolates were collected from oak, and three isolates were collected from Norway spruce. No isolates were obtained from the beech wooden fragments (Table 2).

**Table 2.** Number of isolates of Ophiostomatales fungi obtained from “wood traps” buried in the soil of 40 stands in this study.

Taxon no.	Fungus species	Forest stands dominated by			
		*Quercusrobur*	*Pinussylvestris*	*Piceaabies*	*Fagussylvatica*
1	*Heinzbutiniagrandicarpa*	3			
2	*Leptographiumprocerum*		263		
3	*Leptographiumradiaticola*		35		
4	*Ophiostomapiliferum*		7		
5	*Ophiostomaquercus*	10			
6	*Sporothrixbrunneoviolacea*	53			
7	*Sporothrixdentifunda*	11			
8	*Sporothrixeucastaneae*	1			
9	*Sporothrixroztoczensis* sp. nov.		66		
10	*Sporothrixsilvicola* sp. nov.	1	170	3	
	Total no. of isolates	79	541	3	
	Total no. of species	6	6	1	
	Number of examined fragments	600	600	600	600

Based on morphological observations, the fungal isolates obtained from this study were arranged into 10 species. Five fungal species were isolated from pine while six species were isolated from oak fragments. Only one species (designated as taxon 10; Table 2) was isolated from pine, oak, and spruce samples. The most frequently isolated fungi were taxon 2 and taxon 10 represented by 263 and 174 isolates, respectively. The third most abundant fungus was named as taxon 9, which was isolated 66 times. In addition, 53 isolates of taxon 6 were gathered from buried oak branches (Table 2).

### ﻿DNA sequence data and phylogenetic analysis

**Figure 1.**
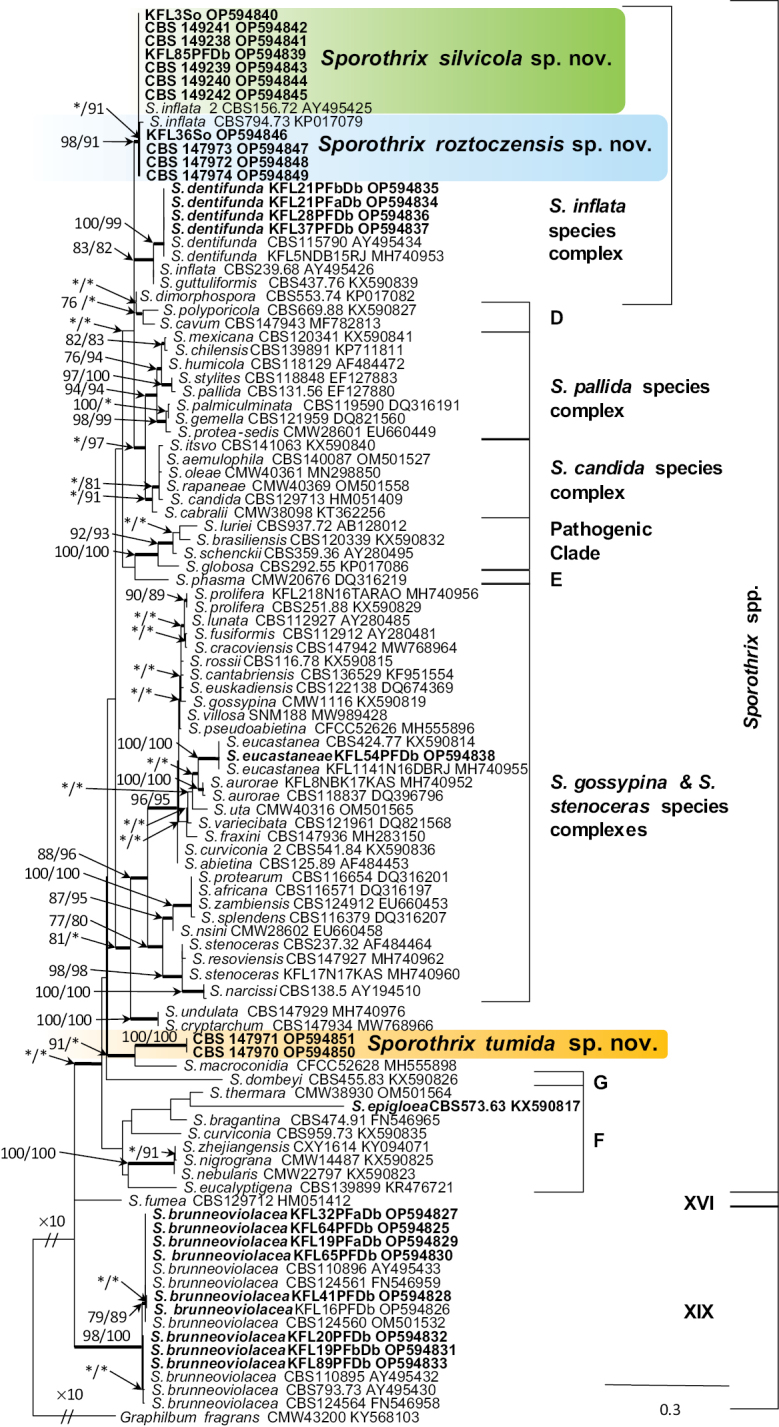
Phylogram from Maximum Likelihood (ML) analyses of ITS data for *Sporothrix* spp. Sequences obtained in this study are in bold. Bootstrap values (if ≥ 75%) for ML and Maximum Parsimony (MP) analyses are presented at the nodes as follows: ML/MP. Bold branches indicate posterior probabilities values ≥ 0.95 obtained from Bayesian Inference (BI) analyses. * Bootstrap values < 75%. The tree is drawn to scale (see bar) with branch lengths measured in the number of substitutions per site. *Graphilbumfragrans* represents the outgroup.

Based on analysis of ITS and LSU sequence data, of the 623 isolates collected in this study, 305, 298, 17 and 3 isolates resided in *Sporothrix* (Fig. 1), *Leptographium* (Suppl. material 2: fig. S3), *Ophiostoma* and *Heinzbutinia* (Suppl. material 2: fig. S4), respectively. Most of the isolates belonging to *Leptographium* grouped in the *L.procerum* species complex, while most of the isolates belonging to *Sporothrix* nested in the *S.inflata* species complex. Phylogenetic analyses of these datasets separated the isolates into 11 distinct taxa, eight of which were previously described species and three represented novel species.

In the genus *Heinzbutinia*, analyses of *TUB*2 sequences data (Suppl. material 2: fig. S5) showed that taxon 1 belonged to *Heinzbutiniagrandicarpa* (Kowalski & Butin) Z.W. de Beer & M. Procter. In *Leptographium* genus, taxon 2 was represented by nine isolates grouping in the *L.procerum* species complex (Suppl. material 2: fig. S3) and *TUB*2 and *TEF*1 sequence analyses confirmed this taxon was conspecific with *L.procerum* (W.B. Kendr.) M.J. Wingf. (Suppl. material 2: figs S6, S7). Taxon 3 was represented by four isolates that grouped in the *L.galeiforme* species complex (Suppl. material 2: fig. S3) and *TUB*2 and *TEF*1 sequence analyses confirmed that these isolates represented *L.radiaticola* (J.J. Kim, Seifert & G.H. Kim) M. Procter & Z.W. de Beer (Suppl. material 2: figs S8, S9).

In the genus *Ophiostoma* taxon 4 was represented by two isolates that did not group in any species complex (Suppl. material 2: fig. S4). Analyses of *TUB*2 sequence data (sSuppl. material 2: fig. S5) showed that this taxon belongs to *O.piliferum* (Fr.) Syd. & P. Syd. Taxon 5 was represented by four isolates in the *O.ulmi* species complex (Suppl. material 2: fig. S4), while *TUB*2 sequences grouped this taxon with *O.quercus* (Georgev.) Nannf. (Suppl. material 2: fig. S5).

**Figure 2.**
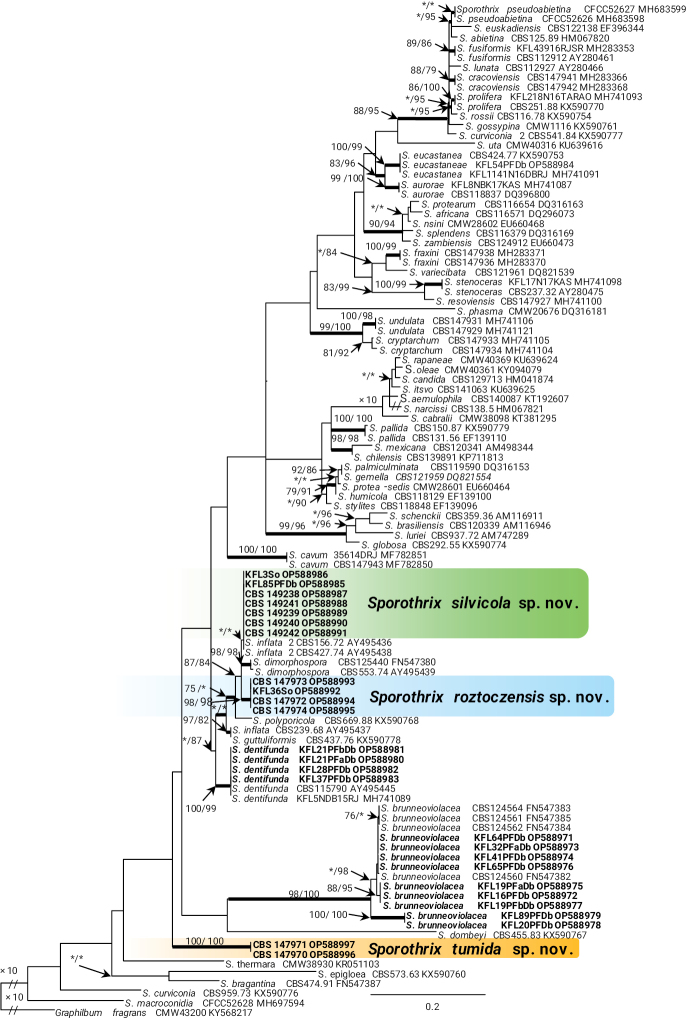
Phylogram from Maximum Likelihood (ML) analyses of *TUB*2 data for *Sporothrix* spp. Sequences obtained in this study are in bold. Bootstrap values (if ≥ 75%) for ML and Maximum Parsimony (MP) analyses are presented at the nodes as follows: ML/MP. Bold branches indicate posterior probabilities values ≥ 0.95 obtained from Bayesian Inference (BI) analyses. * Bootstrap values < 75%. The tree is drawn to scale (see bar) with branch lengths measured in the number of substitutions per site. *Graphilbumfragrans* represents the outgroup.

**Figure 3.**
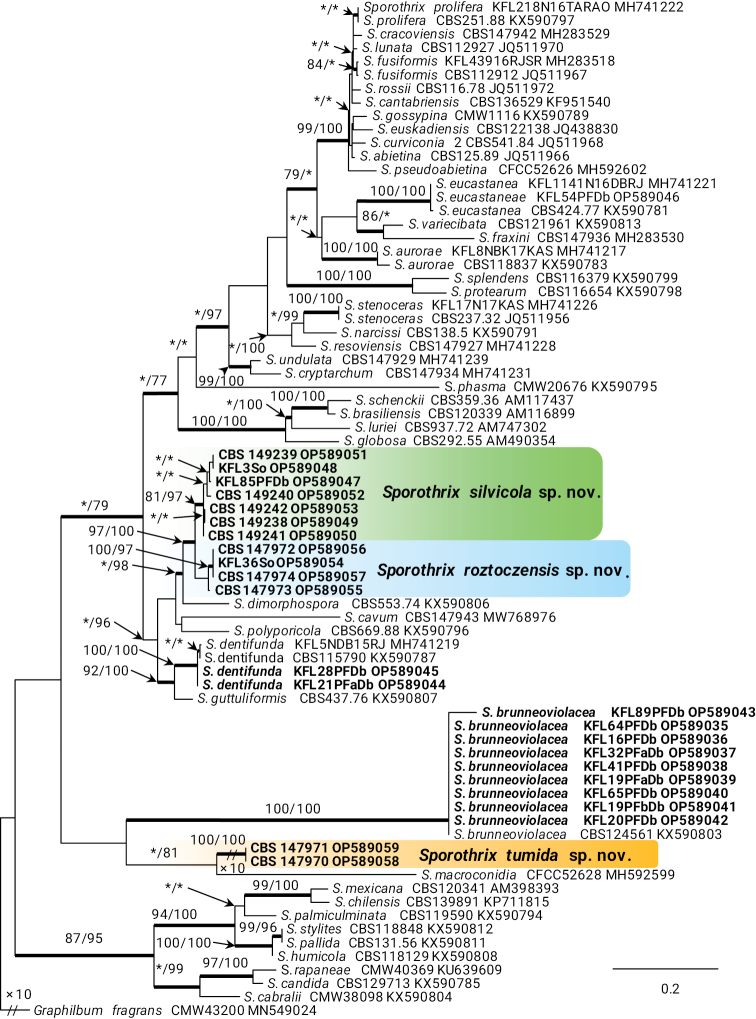
Phylogram from Maximum Likelihood (ML) analyses of *CAL* data for *Sporothrix* spp. Sequences obtained in this study are in bold. Bootstrap values (if ≥ 75%) for ML and Maximum Parsimony (MP) analyses are presented at the nodes as follows: ML/MP. Bold branches indicate posterior probabilities values ≥ 0.95 obtained from Bayesian Inference (BI) analyses. * Bootstrap values < 75%. The tree is drawn to scale (see bar) with branch lengths measured in the number of substitutions per site. *Graphilbumfragrans* represents the outgroup.

**Figure 4.**
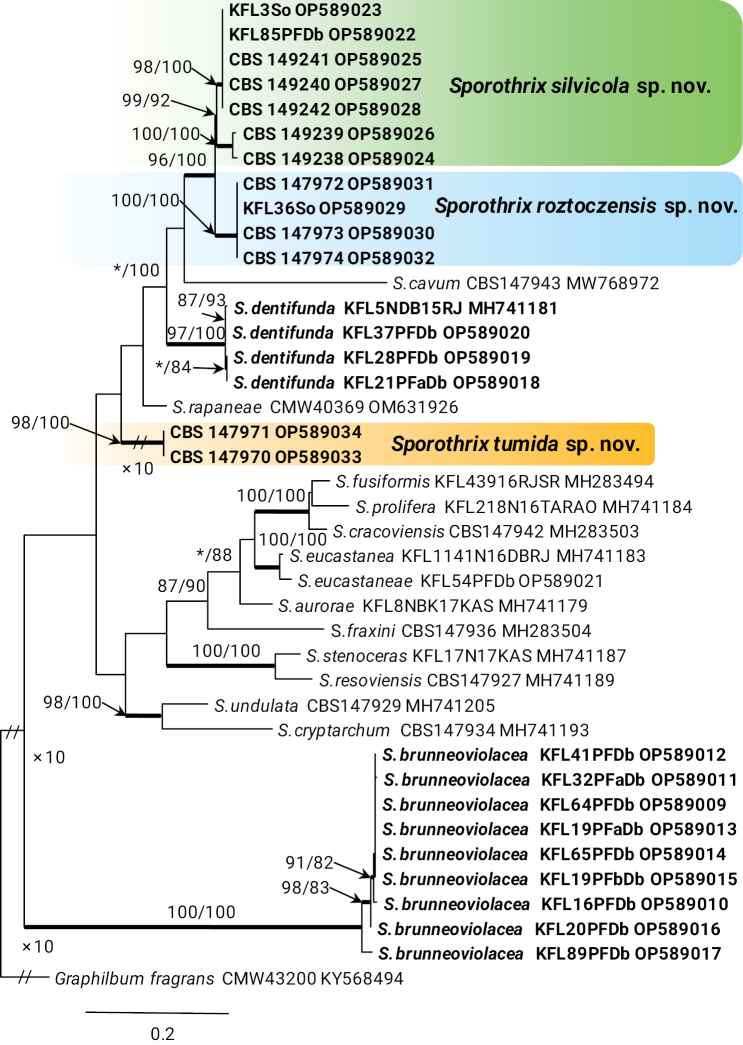
Phylogram from Maximum Likelihood (ML) analyses of *TEF*1 data for the *Sporothrix* spp. Sequences obtained in this study are in bold. Bootstrap values (if ≥ 75%) for ML and Maximum Parsimony (MP) analyses are presented at the nodes as follows: ML/MP. Bold branches indicate posterior probabilities values ≥ 0.95 obtained from Bayesian Inference (BI) analyses. * Bootstrap values < 75%. The tree is drawn to scale (see bar) with branch lengths measured in the number of substitutions per site. *Graphilbumfragrans* represents the outgroup.

**Figure 5.**
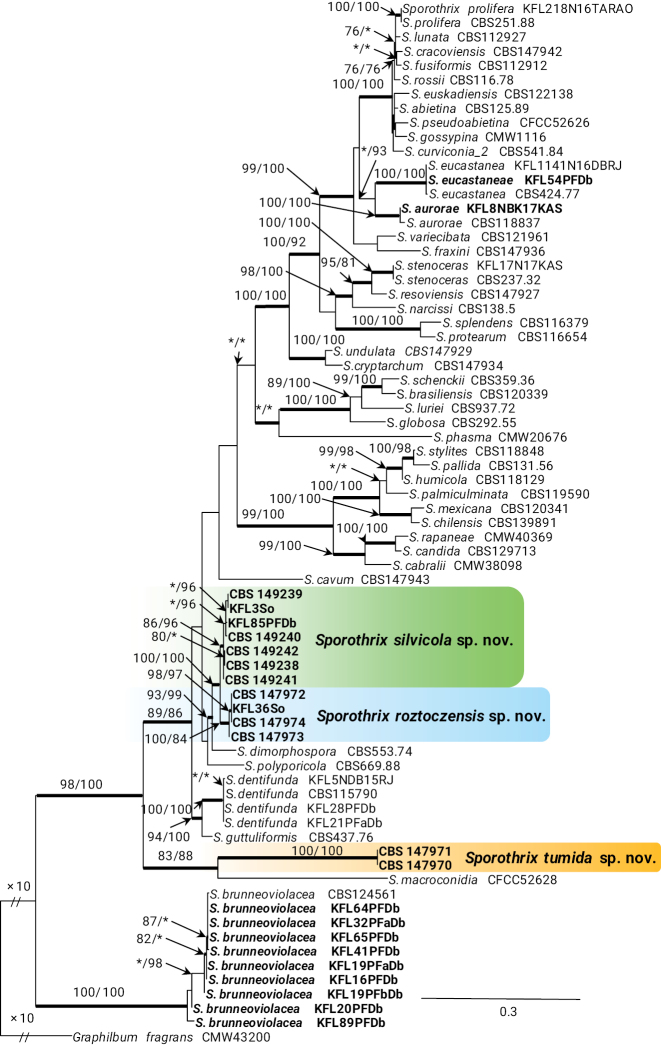
Phylogram from Maximum Likelihood (ML) analyses of the combined datasets of ITS+*BT*+*CAL* for *Sporothrix* spp. Sequences obtained in this study are in bold. Bootstrap values (if ≥ 75%) for ML and Maximum Parsimony (MP) analyses are presented at the nodes as follows: ML/MP. Bold branches indicate posterior probabilities values ≥ 0.95 obtained from Bayesian Inference (BI) analyses. * Bootstrap values < 75%. The tree is drawn to scale (see bar) with branch lengths measured in the number of substitutions per site. *Graphilbumfragrans* represents the outgroup.

In the genus *Sporothrix*, the four isolates of taxon 7 resided in the *S.inflata* species complex and grouped with the ex-type isolate of *S.dentifunda* (Aghayeva & M.J. Wingf.) Z.W. de Beer, T.A. Duong & M.J. Wingf. based on the ITS, *TUB*2, *CAL*, and *TEF*1 phylogenies (Figs 1–4). Taxa 9 and 10 also belonged to the *S.inflata* species complex (Fig. 1) as defined by de Beer et al. (2022) and were represented by four and seven isolates, respectively. Based on the *TUB*2 phylogeny, taxon 9 was close to *S.dimorphospora* and ‘*S.inflata* 2’ and formed a distinct and well-supported clade, while taxon 10 formed a distinct and well-supported clade which included isolates of ‘*S.inflata* 2’ (Fig. 2). Based on the *CAL* phylogeny, taxa 9 and 10 formed two distinct and well-supported clades which were close to, but distinct from *S.dimorphospora* (Fig. 3). Based on the *TEF*1 sequence data (Fig. 4), both taxa formed distinct and well-supported clades, and thus represented novel species. This was supported by the combined analyses of the ITS, *TUB*2 and *CAL* datasets (Fig. 5). Taxon 8 was represented by one isolate and grouped in the *S.gossypina* & *S.stenoceras* species complexes (Fig. 1). *TUB*2, *CAL*, and *TEF*1 phylogenies (Figs 2–4) showed that this taxon is *S.eucastaneae* (R.W. Davidson) Z.W. de Beer, T.A. Duong & M.J. Wingf. Taxon 11 was represented by two isolates collected from fallen pine shoots and did not group in any species complex (Fig. 1). The combined analyses of the ITS, *TUB*2, and *CAL* datasets (Fig. 5) showed that this taxon formed a distinct and well-supported clade which was closest to, but clearly distinct from *S.macroconidia* H.M. Wang, Q. Lu & Zhen Zhang, and thus represented a novel species.

Taxon 6 was represented by nine isolates grouped separately from *Sporothrix* and belonged to lineage XIX (Fig. 1) as defined by de Beer et al. (2022). Analyses of *TUB*2 and *CAL* sequences data (Figs 2, 3) showed that this taxon is *Sporothrixbrunneoviolacea*.

### ﻿Taxonomy

#### ﻿ Sporothrix roztoczensis

Taxon classificationFungiOphiostomatalesOphiostomataceae

R. Jankowiak & P. Bilański sp. nov.

D965ECFC-5911-5E5F-B542-053283FD52EB

 845660

Fig. 6

##### Etymology.

Referring to the highland (from Polish: Roztocze) located in eastern Poland where this fungus was collected.

**Figure 6.**
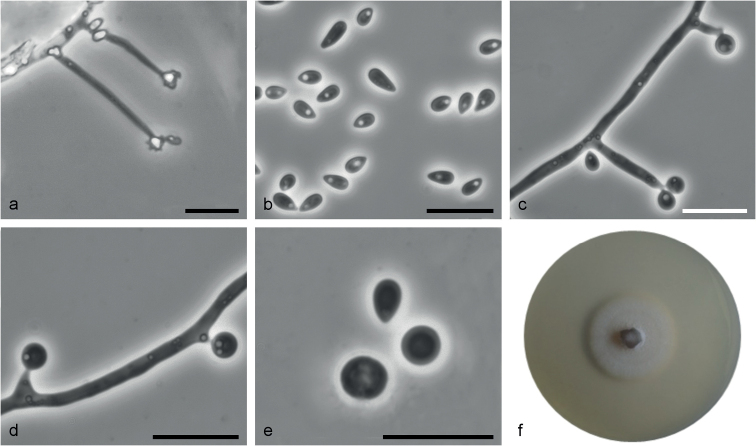
*Sporothrixroztoczensis* sp. nov. (CBS 147973) **a** conidiogenous cell with an inflated cluster of denticles at the apex **b** conidia **c** globose conidia arising on conidiophore **d** globose conidia arising on denticles formed directly from hyphae **e** globose conidia **f** fourteen-day-old culture on MEA. Scale bars: 10 μm.

##### Diagnosis.

*Sporothrixroztoczensis* differs from the phylogenetically closely related species *S.dimorphospora* and *S.silvicola* with respect to its conidia dimensions.

##### Type.

Poland, Lubelskie Province, Józefów, from wood buried in soil under 58-year- old managed *Pinussylvestris* forest, July 2015, *Ł. Chyrzyński* (O-F-259436 ***holotype***, culture ex-type CBS 147973).

##### Description.

Sexual morph not observed. Asexual structures produced on sterilized Scots pine twigs placed on the surface of malt agar in Petri dishes. ***Conidiophores*** hyaline, one-celled, micronematous, simple or branched, either borne on vegetative hyphae or on upright hyphae. ***Conidiogenous cells*** blastic, cylindrical, terminal, lateral or intercalary, straight or curved, constricted at the base and tapering towards the apex, (2.3–)6.6–32.8(–50.5) μm long, (0.6–)1.1–1.6(–2) μm wide at the base, apical part forming conidia by sympodial proliferation on swollen a cluster of conidium-bearing denticles, (0.9–)1.6–3.3(–5) μm long and (1–)1.9–3.9(–6.2) μm wide, denticles very seldom arise below the swollen cluster. ***Conidia*** of two types: 1) abundant in cultures, hyaline, unicellular, smooth, ellipsoid, guttuliform, pointed at the base, sometimes curved (2.5–)3.2–5.1(–7) × (1.4–)1.6–2.1(–2.5) μm, formed directly on denticles; 2) abundant in cultures, subhyaline to lightly pigmented, unicellular, globose to subglobose, sometimes pointed at the base, (2.5–)2.9–3.6(–4.1) μm in diameter, formed singly, on lateral or intercalary conidiogenous cells or denticles directly emerging from vegetative hyphae.

##### Culture characteristics.

Colonies with optimal growth at 20 °C on 2% MEA reaching an average of 31.3 mm (± 3.98 mm) after 14 days, with a radial growth rate of 0.87 (± 0.14) mm/d, growth somewhat slower at 15 °C (26.3 mm diameter), no growth at 30 and 35 °C; white gray, floccose, flat, growing in a circular pattern with entire margins.

##### Distribution.

Known only from the type location (Poland).

##### Additional specimen examined.

Poland, Lubelskie Province, Józefów, from wood buried in soil under 88-year-old managed *Pinussylvestris* forest, July 2015, *Ł. Chyrzyński* (O-F-259435, culture CBS 147972).

##### Notes.

This species is phylogenetically distinct from the other *Sporothrix* species based on the *TUB*2, *CAL*, and *TEF*1 sequences. *Sporothrixroztoczensis* is closely related to *S.dimorphospora*, and *S.silvicola* sp. nov. *Sporothrixsilvicola* has larger sympodial conidia (3.2–10.4 × 1.4–3.6 μm) compared with *S.dimorphospora* (3–8 × 1.5–3 μm, Madrid et al. 2010) and *S.roztoczensis* (2.5–7 × 1.4–2.5 μm). In addition, denticles in *S.silvicola* arise abundantly below the swollen cluster compared with other species, where denticles are limited to the apical cluster. Also the shape of pigmented conidia differed. In *S.roztoczensis* they are globose or subglobose while more obovoid in *S.dimorphospora* and *S.silvicola*. Conidia of *S.roztoczensis* are smaller (2.5–4.1 μm in diam.) compared to *S.dimorphospora* (3–5 × 3.5 μm) and *S.silvicola* (2.6–4.8 × 1.4–3.9 μm). In addition, *S.roztoczensis* rarely produced intercalary conidiogenous cells, which are commonly found in culture of *S.silvicola*.

#### ﻿ Sporothrix silvicola

Taxon classificationFungiOphiostomatalesOphiostomataceae

R. Jankowiak & P. Bilański sp. nov.

D33B212F-5554-595C-8399-4D02840F48A7

 845658

Fig. 7

##### Etymology.

Referring to the Latin *silva* (forest) and –*cola* (inhabiting), with reference to its woody habitat.

**Figure 7.**
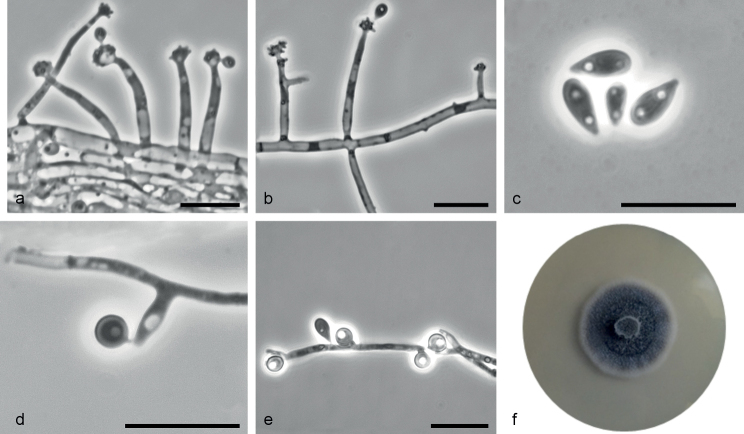
*Sporothrixsilvicola* sp. nov. (CBS 149241) **a, b** conidiogenous cell with an inflated cluster of denticles at the apex **c** conidia **d** globose conidia arising on conidiophore **e** globose conidia arising on denticles formed directly from hyphae **f** fourteen-day-old culture on MEA. Scale bars: 10 μm.

##### Diagnosis.

*Sporothrixsilvicola* differs from the phylogenetically closely related species *S.dimorphospora* and *S.roztoczensis* with respect to its conidia dimensions.

##### Type.

Poland, Lubelskie Province, Józefów, from wood buried in soil under 43-year- old managed *Pinussylvestris* forest, July 2015, *Ł. Chyrzyński*, (O-F-259451 ***holotype***, culture ex-type CBS 149241).

##### Description.

Sexual morph not observed. Asexual structures produced on sterilized Scots pine twigs placed on the surface of malt agar in Petri dishes. ***Conidiophores*** hyaline, one-celled, micronematous, simple, either borne on vegetative hyphae or on upright hyphae. ***Conidiogenous cells*** blastic, cylindrical, terminal, lateral or intercalary, straight or curved, constricted at the base and tapering towards the apex, (2.2–)11.6–35.6(–60.5) μm long, (0.7–)1–1.5(–1.8) μm wide at the base, apical part forming conidia by sympodial proliferation on swollen cluster of conidium-bearing denticles, (1.4–)2.6–4.4(–5.5) μm long and (1.5–)2.1–3.4(–4.1) μm wide, denticles often arise below the swollen cluster. ***Conidia*** of two types: 1) abundant in cultures hyaline, unicellular, smooth, guttuliform, ellipsoid, pointed at the base, sometimes curved (3.2–)3.6–6.4(–10.4) × (1.4–)1.6–2.5(–3.6) μm, formed directly on denticles; 2) abundant in cultures, subhyaline to lightly pigmented, unicellular, smooth, subglobose to broadly ellipsoidal, sometimes pointed at the base, (2.6–)3.1–4.1(–4.8) μm × (1.4–)2.1–3.4(–3.9) μm diam., formed singly, on lateral or intercalary conidiogenous cells or denticles directly emerging from vegetative hyphae.

##### Culture characteristics.

Colonies with optimal growth at 20 °C on 2% MEA reaching an average of 32 mm (± 1.86 mm) after 14 days, with radial growth rate 0.89 (± 0.07) mm/d, growth somewhat slower at 15 °C (26.6 mm diameter), no growth at 30 and 35 °C; dark grey to olivaceous with white margins, floccose, lanose with abundant white aerial hyphae, flat, growing in a circular pattern with entire margins.

##### Distribution.

Known only from the type location (Poland).

##### Additional specimen examined.

Poland, Lubelskie Province, Józefów, from wood buried in soil under 93-year old managed *Pinussylvestris* forest, July 2015, *Ł. Chyrzyński* (O-F-259450, culture CBS 149240).

##### Notes.

This species is phylogenetically distinct from the other *Sporothrix* species based on the *TUB*2, *TEF*1, and *CAL* sequences. The morphological differences between *S.dimorphospora* and *S.roztoczensis* are described in the section treating *S.roztoczensis*. *Sporothrixsilvicola* had identical ITS and *TUB*2 sequences as two isolates of ‘*S.inflata* 2’ (CBS 156.72, CBS 427.74) obtained from greenhouse soil and isolated from *Lilium* sp. in the Netherlands (Aghayeva et al. 2005; de Beer et al. 2016).

#### ﻿ Sporothrix tumida

Taxon classificationFungiOphiostomatalesOphiostomataceae

R. Jankowiak & P. Bilański sp. nov.

C100EDB5-89EA-5EF3-8110-7A1E9841BC48

 845661

Fig. 8

##### Etymology.

Referring to the Latin *tumeo* (swollen) to reflect the characteristically inflated hyphae and conidiogenous cells.

**Figure 8.**
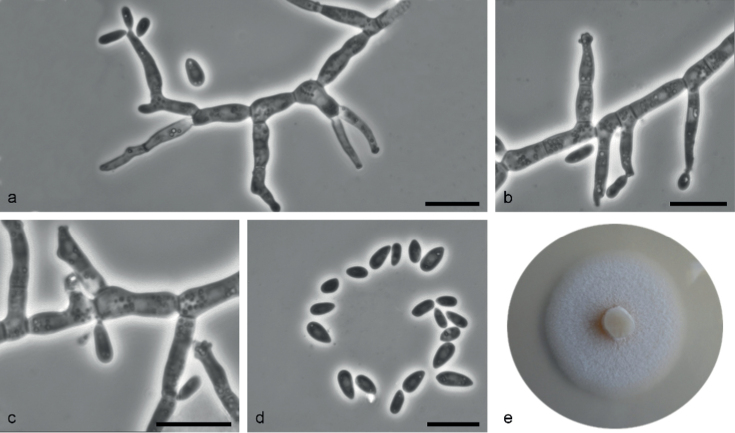
*Sporothrixtumida* sp. nov. (CBS 147970) **a, b** conidiogenous cell with an inflated cluster of denticles at the apex **c** denticles arising directly from hyphae **d** conidia **e** fourteen-day-old culture on MEA. Scale bars: 10 μm.

##### Diagnosis.

*Sporothrixtumida* differs from the phylogenetically closely related *S.macroconidia* in respect of dimensions of its conidia.

##### Type.

Poland, Podkarpackie Province, Mielec, from fallen shoots of *Pinussylvestris* pruned by *Tomicus* sp., October 2007, *P. Bilański*, (O-F-259433 ***holotype***, culture ex-type CBS 147970).

##### Description.

Sexual morph not observed. Asexual structures produced on sterilized Scots pine twigs placed on the surface of malt agar in Petri dishes. ***Conidiophores*** hyaline, one- or two-celled, micronematous, simple or slightly branched, either borne on vegetative hyphae or on upright hyphae, often inflated. ***Conidiogenous cells*** blastic, cylindrical, terminal, straight, constricted at the base and strong tapering towards the apex, (7.8–)12–25.4(–34.7) μm long, (1.3–)1.6–2.6(–3.5) μm wide at the base, apical part forming conidia by sympodial proliferation on swollen a cluster of conidium-bearing faintly developed denticles, (1–)1.2–2.5(–3.3) μm long and (1.1–)1.4–2.9(–4.7) μm wide, denticles sometimes arise directly from hypha. ***Conidia*** abundant in cultures hyaline, unicellular, smooth, guttuliform, ellipsoid, sometimes curved, slightly pointed at the base (3.4–)4.2–6.6(–8.7) × (1.3–)1.9–3.1(–3.9) μm.

##### Culture characteristics.

Colonies with optimal growth at 25 °C on 2% MEA reaching an average of 36.3 mm (± 0.62 mm) after 14 days, with radial growth rate 1.05 (± 0.02) mm/d, growth somewhat slower at 30 °C (29.6 mm diameter); white, flat, floccose, growing in a circular pattern with entire margins.

##### Host tree.

*Pinussylvestris*.

##### Insect vector.

*Tomicus* spp.

##### Distribution.

Known only from the type location (Poland).

##### Additional specimen examined.

Poland, Podkarpackie Province, Mielec, from fallen shoots of Scots pine pruned by *Tomicus* sp., October 2007, *R. Jankowiak*, (O-F-259434, culture CBS 147971).

##### Notes.

This species is phylogenetically distinct from the other *Sporothrix* species based on the ITS, *TUB*2, and *CAL* sequences. *Sporothrixtumida* grouped most closely with *S.macroconidia* (ITS, *CAL*) from which it can also be distinguished by dimensions of conidia (3.4–8.7 × 1.3–3.9 μm vs. 3.6–9.9 × 2.5–9.9 μm, Wang et al. 2019).

## ﻿Discussion

This study reported 10 members of the Ophiostomatales associated with soil under European beech, pedunculate oak, Scots pine, and Norway spruce stands in Poland. Two of these species are newly described here (*Sporothrixroztoczensis* and *S.silvicola*) and were the most abundant species in the forest soil. This demonstrates that there is a rich and poorly studied diversity of species of the Ophiostomatales associated with soil in European forests.

Our results revealed a greater than expected diversity of Ophiostomatales fungi in soil, while confirming that the methods used here (autoclaved branches buried in the soil) are useful for the detection of soil-borne fungi from this order. To date, *Sporothrix* is the main Ophiostomatales genus to be found in soil samples (e.g., de Hoog 1974; de Meyer et al. 2008; Madrid et al. 2010; de Beer et al. 2016; Rodrigues et al. 2017; Ramírez-Soto et al. 2018). *Leptographium* species have also been isolated from soil, although these are found primarily in tree roots (Eckhardt 2003). For example, *Leptographiumwageneri* (W.B. Kendr.) M.J. Wingf., a causative agent of black stain root disease of conifers in the western United States and Canada, can be transmitted between diseased and healthy roots through continuous xylem in root grafts (Landis and Helburg 1976), by short-distance growth through soil (Goheen and Cobb 1978) and by insect vectors (Harrington and Cobb 1988).

The dominant tree species in the stands strongly affected fungal species richness and taxonomic diversity. Most of the fungi were isolated from the pine and oak stands, while only three isolates were obtained from the spruce stands, and no fungi were isolated from the beech stands. *Sporothrixsilvicola* was the only fungal species found in pine-, oak- and spruce-dominated stands, although it was highly abundant only in pine stands. *Leptographiumprocerum* and, to a lesser extent, *L.radiaticola* and *S.roztoczensis*, were also abundant in pine stands. In contrast, wood buried in oak stands was mostly colonized by *S.brunneoviolacea* and less frequently by *S.dentifunda* and *O.quercus*.

This research demonstrated that *Sporothrix* species can be soil-borne, validating previous studies in South Africa (de Meyer et al. 2008), Spain and USA (Madrid et al. 2010). Five of the species collected in this study belong to *Sporothrix*, including the two newly described species. *Sporothrixbrunneoviolacea* (Madrid et al. 2010) and ‘*S.inflata* 2’ (de Hoog 1974; de Beer et al. 2016) were previously reported in soil from Europe, and this study shows that *S.dentifunda* and *S.eucastaneae* also occur in forest soil. The identified *Sporothrix* species showed different affinities to tree hosts, as *S.brunneoviolacea*, *S.dentifunda*, and *S.eucastaneae* were found in oak stands while *S.silvicola* and *S.roztoczensis* were reported in pine stands. This is in congruence with previous reports: *Sporothrixbrunneoviolacea* was already isolated from meadow soil in Germany, from soil under mixed stands in Spain, and from the roots of *Quercus* spp. in Austria (Halmschlager and Kowalski 2003; Madrid et al. 2010). Similarly, *S.dentifunda* has been isolated from the wood of *Quercus* sp. in Poland and Hungary (Aghayeva et al. 2005), as well as from wounds on *Q.robur* in Poland (Jankowiak et al. 2019b). *Sporothrixeucastaneae* has also been previously isolated from oak stands in Poland, where this fungus was associated with oak-infesting bark beetles (Jankowiak et al. 2019a) and wounded oaks (Jankowiak et al. 2019b).

The *Sporothrix* species from pine stands, *S.silvicola* and *S.roztoczensis*, are newly described sister species that reside in the *S.inflata* species complex (de Beer et al. 2022). Although both species inhabited the same environment, they can be distinguished based on phylogenetic analyses and morphological characteristics, such as differences in conidia dimensions and shapes. Both species produced two different conidial types, which is a characteristic that has been found in other *Sporothrix* species, including *Sporothrixdimorphospora* and *S.brunneoviolacea* (Madrid et al. 2010), *S.brasiliensis*, *S.globosa*, and *S.mexicana* (Marimon et al. 2007), as well as *S.cryptarchum* R. Jankowiak & A. Ostafińska and *S.undulata* R. Jankowiak & A. Ostafińska (Ostafińska et al. 2021). In Poland, *S.silvicola* named as ‘*S.inflata* 2’ was also sporadically found in association with *Scolytusintricatus* (Ratzeburg) on *Q.robur* (Jankowiak et al. 2019a) and wounded *Tiliacordata* Mill. (Jankowiak et al. 2019b), suggesting that the fungus may not be limited to conifer-dominated habitats. More surveys should be conducted to determine the range of the fungus, and to test their affinities to pine forests.

Our results also demonstrated that some *Leptographium* species are soil-borne, supporting the findings of Eckhardt (2003) that *L.procerum* is a soil-borne fungus. This species was previously isolated from roots of dying and dead young Scots pines (Jankowiak et al. 2012) and was often found to be carried by root-feeding bark beetles and weevils in Poland (Jankowiak and Bilański 2013a, b, c). A high abundance of this species in soil and pine roots suggests that *L.procerum* may be capable of infecting roots via soil. According to previous studies, *L.procerum* can spread over short distances via root-to-root contact between infected and uninfected host trees, as well as through soil as short term survival in the soil around infected trees has been observed (Lackner and Alexander 1984; Alexander et al. 1988; Jacobs and Wingfield 2001; Eckhardt et al. 2004). In our opinion, the presence of *L.radiaticola* in the soil of pine stands suggests that other *Leptographium* species may be similarly transmitted. Possible transmission through soil has been also observed for *L.wageneri* (Goheen and Cobb 1978). In addition, *L.costaricense* G. Weber, Spaaij & M.J. Wingf. (Weber et al. 1996) and *L.reconditum* Jooste (Jooste 1978) were found in the rhizospheres of *Talaumasambuensis* Pittier and *Triticum*, respectively.

Although *O.piliferum* was rarely isolated in this study, we confirmed that it is soil-borne. Its presence was unsurprising because this species is commonly found staining pine wood in Poland (Jankowiak et al. 2018b, 2021). In addition, *O.piliferum* was also found in soil from sites exposed to different wood preservative types (Kirker et al. 2017). Finally, *O.quercus* was also reported in soil in this study. This globally widespread species (Taerum et al. 2018) is a common wood-infecting fungus associated with many species of bark and wood boring beetles in Poland (Jankowiak et al. 2019a, b), and may be more commonly found in soil with additional surveys.

*Sporothrixtumida* was collected from fallen shoots of Scots pine that were pruned by *Tomicus* species in Poland (Jankowiak and Kolařík 2011). The species is the most closely related to *S.macroconidia*, which was recently described from *Tomicusyunnanensis* Kirkendall & Faccoli and *T.brevipilosus* Eggers on *Pinusyunnanensis* Franch. and *P.kesiya* Royle ex Gordon in south-western China (Wang et al. 2019). The new species identified in this study can be easily distinguished from *S.macroconidia* by phylogenetic analysis and morphological characteristics.

Our work has led to the discovery of three novel *Sporothrix* species, bringing the total number of *Sporothrix* species in Poland to 20. The present study has shown that forest soil under pine and oak stands in Poland is remarkably rich in Ophiostomatales species. Our surveys were conducted in 35–135 year old managed stands, showing that even recently managed forests can house undescribed fungal species. Additional species of these fungi will most likely emerge when more extensive surveys are conducted in other parts of Europe as forest soil fungi are influenced by a variety of biotic and abiotic factors, including climate, soil physicochemical properties, forest age, tree compositions and management type (e.g. Baldrian et al. 2012; Tedersoo et al. 2014; Goldmann et al. 2015; Urbanová et al. 2015). Therefore, future research should focus on identifying soil-borne Ophiostomatales species in forests with different tree compositions and soil characteristics.

## Supplementary Material

XML Treatment for Sporothrix roztoczensis

XML Treatment for Sporothrix silvicola

XML Treatment for Sporothrix tumida

## Supplementary materials

Supplementary material 1 *Pinussylvestris* branches used to bait Ophiostomatales species from soil in this study Data type figures (word document) Explanation note An example of a *Pinussylvestris* branch used to bait Ophiostomatales from soil. *Pinussylvestris* branches after removal from soil. https://binary.pensoft.net/file/851009This dataset is made available under the Open Database License (http://opendatacommons.org/licenses/odbl/1.0/). The Open Database License (ODbL) is a license agreement intended to allow users to freely share, modify, and use this Dataset while maintaining this same freedom for others, provided that the original source and author(s) are credited.Piotr Bilański, Robert Jankowiak, Halvor Solheim, Paweł Fortuna, Łukasz Chyrzyński, Paulina Warzecha, Stephen Joshua Taerum

Supplementary material 2 Phylograms from Maximum Likelihood analyses Data type figures (word document) Explanation note Phylogram from Maximum Likelihood (ML) analyses of LSU data for *Leptographium* spp. Phylogram from Maximum Likelihood (ML) analyses of ITS data for *Ophiostoma* spp. Phylogram from Maximum Likelihood (ML) analyses of *TUB*2 data for the *Ophiostomaulmi* species complex. Phylogram from Maximum Likelihood (ML) analyses of *TUB*2 data for the *Leptographiumprocerum* species complex. Phylogram from Maximum Likelihood (ML) analyses of *TEF*1 data for the *Leptographiumprocerum* species complex. Phylogram from Maximum Likelihood (ML) analyses of *TUB*2 data for the *Leptographiumgaleiforme* species complex. Phylogram from Maximum Likelihood (ML) analyses of *TEF*1 data for the *Leptographiumgaleiforme* species complex. https://binary.pensoft.net/file/851010This dataset is made available under the Open Database License (http://opendatacommons.org/licenses/odbl/1.0/). The Open Database License (ODbL) is a license agreement intended to allow users to freely share, modify, and use this Dataset while maintaining this same freedom for others, provided that the original source and author(s) are credited.Piotr Bilański, Robert Jankowiak, Halvor Solheim, Paweł Fortuna, Łukasz Chyrzyński, Paulina Warzecha, Stephen Joshua Taerum

## References

[B1] Aas T Solheim H Jankowiak R Bilański P Hausner G (2018) Four new *Ophiostoma* species associated with hardwood-infesting bark beetles in Norway and Poland. Fungal Biology 122(12): 1142–1158. 10.1016/j.funbio.2018.08.001 30449352

[B2] Aghayeva DN Wingfield MJ Kirisits T Wingfield BD (2005) *Ophiostomadentifundum* sp. nov. from oak in Europe, characterized using molecular phylogenetic data and morphology. Mycological Research 109(10): 1127–1136. 10.1017/S0953756205003710 16279407

[B3] Alexander SA Horner WE Lewis KJ (1988) *Leptographiumprocerum* as a pathogen of pines. In: Harrington TC Cobb Jr FW (Eds) Leptographium Root Diseases on Conifers.American Phytopathological Society Press, St Paul, Minnesota, 97–112.

[B4] Baldrian P Kolařík M Štursová M Kopecký J Valášková V Větrovský T Žifčáková L Šnajdr J Rídl J Vlček Č Voříšková J (2012) Active and total microbial communities in forest soil are largely different and highly stratified during decomposition. The ISME Journal 6(2): 248–258. 10.1038/ismej.2011.95 21776033 PMC3260513

[B5] Carbone I Kohn LM (1999) A method for designing primer sets for speciation studies in filamentous ascomycetes. Mycologia 91(3): 553–556. 10.1080/00275514.1999.12061051

[B6] Darriba D Taboada GL Doallo R Posada D (2012) jModelTest 2: More models, new heuristics and parallel computing. Nature Methods 9(8): 772. 10.1038/nmeth.2109 PMC4594756 22847109

[B7] de Beer ZW Wingfield MJ (2013) Emerging lineages in the Ophiostomatales. In: Seifert KA De Beer ZW Wingfield MJ (Eds) The Ophiostomatoid Fungi: Expanding Frontiers.Centraalbureau voor Schimmelcultures (CBS), Utrecht, The Netherlands, CBS Biodiversity Series 12: 21–46.

[B8] de Beer ZW Harrington TC Vismer HF Vingfield BD Wingfield MJ (2003) Phylogeny of the *Ophiostomastenoceras*–*Sporothrixschenckii* complex. Mycologia 95(3): 434–441. 10.1080/15572536.2004.11833088 21156632

[B9] de Beer ZW Seifert KA Wingfield MJ (2013a) The ophiostomatoid fungi: their dual position in the Sordariomycetes. In: Seifert KA De Beer ZW Wingfield MJ (Eds) The Ophiostomatoid Fungi: Expanding Frontiers.Centraalbureau voor Schimmelcultures (CBS), Utrecht, The Netherlands, CBS Biodiversity Series 12: 1–19.

[B10] de Beer ZW Seifert KA Wingfield MJ (2013b) A nomenclator for ophiostomatoid genera and species in the Ophiostomatales and Microascales. In: Seifert KA De Beer ZW Wingfield MJ (Eds) The Ophiostomatoid Fungi: Expanding Frontiers.Centraalbureau voor Schimmelcultures (CBS), Utrecht, The Netherlands, CBS Biodiversity Series 12: 245–322.

[B11] de Beer ZW Duong TA Wingfield MJ (2016) The divorce of *Sporothrix* and *Ophiostoma*: Solution to a problematic relationship. Studies in Mycology 83(1): 165–191. 10.1016/j.simyco.2016.07.001 27616802 PMC5007658

[B12] de Beer W Procter M Wingfield MJ Marincowitz S Duong TA (2022) Generic boundaries in the Ophiostomatales reconsidered and revised. Studies in Mycology 101(1): 57–120. 10.3114/sim.2022.101.02 36059894 PMC9365045

[B13] de Hoog GS (1974) The genera *Blastobotrys*, *Sporothrix*, *Calcarisporium* and *Calcarisporiella* gen. nov. Studies in Mycology 7: 1–84.

[B14] de Meyer EM de Beer ZW Summerbell RC Moharram AM de Hoog GS Vismer HF Wingfield MJ (2008) Taxonomy and phylogeny of new wood- and soil-inhabiting *Sporothrix* species in the *Ophiostomastenoceras*–*Sporothrixschenckii* complex. Mycologia 100(4): 647–661. 10.3852/07-157R 18833758

[B15] Eckhardt LG (2003) Biology and Ecology of *Leptographium* species and their vectors as components of loblolly Pine Decline. PhD Thesis, Louisiana State University, Bâton-Rouge, USA.

[B16] Eckhardt LG Jones JP Klepzig KD (2004) Pathogenicity of *Leptographium* species associated with loblolly pine decline. Plant Disease 55(11): 1174–1178. 10.1094/PDIS.2004.88.11.1174 30795310

[B17] Ekici A Chadburn S Chaudhary N Hajdu LH Marmy A Peng S Boike J Burke E Friend AD Hauck C Krinner G Lange M Miller PA Beer C (2014) Site-level model intercomparison of high latitude and high altitude soil thermal dynamics in tundra and barren landscapes. The Cryosphere Discussion 8: 4959–5013. 10.5194/tcd-8-4959-2014

[B18] Gardes M Bruns TD (1993) ITS primers with enhanced speciﬁcity for basidiomycetes: Application to the identiﬁcation of mycorrhizae and rusts. Molecular Ecology 2(2): 113–118. 10.1111/j.1365-294X.1993.tb00005.x 8180733

[B19] Glass NL Donaldson GC (1995) Development of primer sets designed for use with the PCR to amplify conserved genes from filamentous ascomycetes. Applied and Environmental Microbiology 61(4): 1323–1330. 10.1128/aem.61.4.1323-1330.1995 7747954 PMC167388

[B20] Goheen DJ Cobb FW (1978) Occurrence of *Verticicladiellawageneri* and its perfect state, *Ceratocystiswageneri* sp. nov., in insect galleries. Phytopathology 68(8): 1192–1195. 10.1094/Phyto-68-1192

[B21] Goldmann K Schöning I Buscot F Wubet T (2015) Forest management type influences diversity and community composition of soil fungi across temperate forest ecosystems. Frontiers in Microbiology 6: e1300. 10.3389/fmicb.2015.01300 PMC4656839 26635766

[B22] Grobbelaar JW Aghayeva DN De Beer ZW Bloomer P Wingfield MJ Wingfield BD (2009) Delimitation of *Ophiostomaquercus* and its synonyms using multiple gene phylogenies. Mycological Progress 8(3): 221–236. 10.1007/s11557-009-0594-4

[B23] Guindon S Gascuel O (2003) A simple, fast and accurate method to estimate large phylogenies by maximum-likelihood. Systematic Biology 52(5): 696–704. 10.1080/10635150390235520 14530136

[B24] Guindon S Dufayard JF Lefort V Anisimova M Hordijk W Gascuel O (2010) New algorithms and methods to estimate maximum-likelihood phylogenies: Assessing the performance of PhyML 3.0. Systematic Biology 59(3): 307–321. 10.1093/sysbio/syq010 20525638

[B25] Hall TA (1999) BioEdit: a user-friendly biological sequence alignment editor and analysis program for Windows 95/98/NT. Nucleic Acids Symposium 41: 95–98.

[B26] Halmschlager E Kowalski T (2003) *Sporothrixinflata*, a root-inhabiting fungus of *Quercusrobur* and *Q.petrea*. Mycological Progress 2(4): 259–266. 10.1007/s11557-006-0063-2

[B27] Harrington TC Cobb FW (1988) *Leptographium* Root Diseases on Conifers. APS Press, St. Paul, 147 pp.

[B28] Jacobs K Wingfield MJ (2001) *Leptographium* Species: Tree Pathogens, Insect Associates, and Agents of Blue-Stain. APS Press, St. Paul, 207 pp.

[B29] Jacobs K Bergdahl DR Wingfield MJ Halik S Seifert KA Bright DE Wingfield BD (2004) *Leptographiumwingfieldii* introduced into North America and found associated with exotic *Tomicuspiniperda* and native bark beetles. Mycological Research 108(4): 411–418. 10.1017/S0953756204009748 15209281

[B30] Jankowiak R Bilański P (2013a) Ophiostomatoid fungi associated with root-feeding bark beetles in Poland. Forest Pathology 43(5): 422–428. 10.1111/efp.12049

[B31] Jankowiak R Bilański P (2013b) Association of the pine-infesting *Pissodes* species with ophiostomatoid fungi in Poland. European Journal of Forest Research 132(3): 523–534. 10.1007/s10342-013-0693-2

[B32] Jankowiak R Bilański P (2013c) Diversity of ophiostomatoid fungi associated with the large pine weevil, *Hylobiusabietis*, and infested Scots pine seedlings in Poland. Annals of Forest Science 70(4): 391–402. 10.1007/s13595-013-0266-z

[B33] Jankowiak R Kolařík M (2010) Diversity and pathogenicity of ophiostomatoid fungi associated with *Tetropium* species colonizing *Piceaabies* in Poland. Folia Microbiologica 55(2): 145–154. 10.1007/s12223-010-0022-9 20490757

[B34] Jankowiak R Kolařík M (2011) Ophiostomatoid fungi isolated from fallen shoots of Scots pine pruned by *Tomicus* species in Poland. Acta Mycologica 46(2): 201–210. 10.5586/am.2011.013

[B35] Jankowiak R Bilański P Kolařik M Wasiuta D (2012) Root-colonizing ophiostomatoid fungi associated with dying and dead young Scots pine in Poland. Forest Pathology 42(6): 492–500. 10.1111/j.1439-0329.2012.00783.x

[B36] Jankowiak R Strzałka B Bilański P Kacprzyk M Lukášová K Linnakoski R Matwiejczuk S Misztela M Rossa R (2017) Diversity of Ophiostomatales species associated with conifer-infesting beetles in the Western Carpathians. European Journal of Forest Research 136(5–6): 939–956. 10.1007/s10342-017-1081-0

[B37] Jankowiak R Ostafińska A Aas T Solheim H Bilański P Linnakoski R Hausner G (2018a) Three new *Leptographium* spp. (Ophiostomatales) infecting hardwood trees in Norway and Poland. Antonie van Leeuwenhoek 111(12): 2323–2347. 10.1007/s10482-018-1123-8 29980901 PMC6245115

[B38] Jankowiak R Bilański P Chyrzyński P Strzałka B (2018b) Identification of sapstain fungi from Scots pine pallets and assessment of their staining ability. European Journal of Plant Pathology 150(2): 307–322. 10.1007/s10658-017-1279-5

[B39] Jankowiak R Strzałka B Bilański P Kacprzyk M Wieczorek P Linnakoski R (2019a) Ophiostomatoid fungi associated with hardwood-infesting bark and ambrosia beetles in Poland: Taxonomic diversity and vector specificity. Fungal Ecology 39: 152–167. 10.1016/j.funeco.2019.02.001

[B40] Jankowiak R Bilański P Ostafińska A Linnakoski R (2019b) Ophiostomatales associated with wounds on hardwood trees in Poland. Plant Pathology 68(7): 1407–1424. 10.1111/ppa.13061

[B41] Jankowiak R Bilański P Strzałka B Linnakoski R Bosak A Hausner G (2019c) Four new *Ophiostoma* species associated with conifer- and hardwood-infesting bark and ambrosia beetles from Czech Republic and Poland. Antonie van Leeuwenhoek 112(10): 1501–15021. 10.1007/s10482-019-01277-5 31140027 PMC6748885

[B42] Jankowiak R Solheim H Bilański P Marincowitz S Wingfield MJ (2020) Seven new species of *Graphilbum* from conifers in Norway, Poland, and Russia. Mycologia 112(6): 1240–1262. 10.1080/00275514.2020.1778375 32634330

[B43] Jankowiak R Szewczyk G Bilański P Jazłowiecka D Harabin B Linnakoski R (2021) Blue-stain fungi isolated from freshly felled Scots pine logs in Poland, including *Leptographiumsosnaicola* sp. nov. Forest Pathology 51(2): e12672. 10.1111/efp.12672

[B44] Jooste WJ (1978) *Leptographiumreconditum* sp.nov. and observations on conidiogenesis in *Verticicladiella*. Transactions of the British Mycological Society 70(1): 152–155. 10.1016/S0007-1536(78)80189-2

[B45] Katoh K Standley DM (2013) MAFFT multiple sequence alignment software version 7, improvements in performance and usability. Molecular Biology and Evolution 30(4): 772–780. 10.1093/molbev/mst010 23329690 PMC3603318

[B46] Kirker GT Bishell AB Jusino MA Palmer JM Hickey WJ Lindner DL (2017) Amplicon-Based Sequencing of Soil Fungi from Wood Preservative Test Sites. Frontiers in Microbiology 8: e1997. 10.3389/fmicb.2017.01997 PMC5651271 29093702

[B47] Kornerup A Wanscher JH (1978) Methuen Handbook of Colour (3^rd^ edn). Eyre Methuen, London, 252 pp.

[B48] Lackner AL Alexander SA (1984) Incidence and development of *Verticicladiellaprocera* in Virginia Christmas tree plantations. Plant Disease 68(3): 210–212. 10.1094/PD-69-210

[B49] Landis TD Helburg LB (1976) Black stain root disease of pinyon pine in Colorado. The Plant Disease Reporter 60: 713–717.

[B50] Linnakoski R Jankowiak R Villari C Kirisits T Solheim H de Beer ZW Wingfield MJ (2016) The *Ophiostomaclavatum* species complex: A newly defined group in the Ophiostomatales including three novel taxa. Antonie van Leeuwenhoek 109(7): 987–1018. 10.1007/s10482-016-0700-y 27142088

[B51] Lòpez-Romero E Reyes-Montes MdR Pèrez-Torres A Ruiz-Baca E Villagómez-Castro JC Mora-Montes HM Flores-Carreón A Toriello C (2011) *Sporothrixschenckii* complex and sporotrichosis, an emerging health problem. Future Microbiology 6(1): 85–102. 10.2217/fmb.10.157 21162638

[B52] Madrid H Gene J Cano J Silvera C Guarro J (2010) *Sporothrixbrunneoviolacea* and *Sporothrixdimorphospora*, two new members of the *Ophiostomastenoceras*–*Sporothrixschenckii* complex. Mycologia 102(5): 1193–1203. 10.3852/09-320 20943519

[B53] Marimon R Cano J Gene J Sutton DA Kawasaki M Guarro J (2007) *Sporothrixbrasiliensis*, *S.globosa*, and *S.mexicana*, three new *Sporothrix* species of clinical interest. Journal of Clinical Microbiology 45(10): 3198–3206. 10.1128/JCM.00808-07 17687013 PMC2045377

[B54] Marincowitz S Duong TA de Beer ZW Wingfield MJ (2015) *Cornuvesica*: A little known mycophilic genus with a unique biology and unexpected new species. Fungal Biology 119(7): 615–630. 10.1016/j.funbio.2015.03.007 26058537

[B55] Musvuugwa T de Beer ZW Duong TA Dreyer LL Oberlander K Roets F (2016) Wounds on *Rapaneamelanophloeos* provide habitat for a large diversity of Ophiostomatales including four new species. Antonie van Leeuwenhoek 109(6): 877–894. 10.1007/s10482-016-0687-4 27022984

[B56] Neto JOM Mello CR da Silva AM de Mello JM Viola MR Yanagi SNM (2017) Temporal stability of soil moisture under effect of three spacings in a eucalyptus stand. Acta Scientiarum. Agronomy 39(3): 393–399. 10.4025/actasciagron.v39i3.32656

[B57] Novotný D Šrůtka P (2004) *Ophiostomastenoceras* and *O.grandicarpum* (Ophiostomatales), first records in the Czech Republic. Czech Mycology 56(1–2): 19–32. 10.33585/cmy.56102

[B58] O’Donnell K Kistler HC Cigelnik E Ploetz RC (1998) Multiple evolutionary origins of the fungus causing Panama disease of banana: Concordant evidence from nuclear and mitochondrial gene genealogies. Proceedings of the National Academy of Sciences of the United States of America 95(5): 2044–2049. 10.1073/pnas.95.5.2044 9482835 PMC19243

[B59] O’Donnell K Nirenberg H Aoki T Cigelnik E (2000) A multigene phylogeny of the *Gibberellafujikuroi* species complex: Detection of additional phylogenetically distinct species. Mycoscience 41(1): 61–78. 10.1007/BF02464387

[B60] Ostafińska A Jankowiak R Bilański P Solheim H Wingfield MJ (2021) Six new species of *Sporothrix* from hardwood trees in Poland. MycoKeys 82: 1–32. 10.3897/mycokeys.82.66603 34393590 PMC8357686

[B61] Ostrowska A Gawliński S Szczubiałka Z (1991) Methods of Analysis and Assessment of Soil and Plant Properties. Institute of Environmental Protection, Warszawa.

[B62] Rambaut A Drummond AJ (2007) Tracer v1.4. http://beast.bio.ed.ac.uk/Tracer

[B63] Ramírez-Soto MC Aguilar-Ancori EG Tirado-Sánchez A Bonifaz A (2018) Ecological Determinants of Sporotrichosis Etiological Agents. Journal of Fungi 4(3): 1–95. 10.3390/jof4030095 PMC6162718 30103554

[B64] Rodrigues AM Fernandes GF Camargo ZP (2017) Sporotrichosis. In: Bayry J (Ed.) Emerging and Re-emerging Infectious Diseases of Livestock.Springer International Publishing AG, Cham, Switzerland, 391–424. 10.1007/978-3-319-47426-7_19

[B65] Ronquist F Huelsenbeck JP (2003) MrBayes 3: Bayesian phylogenetic inference under mixed models. Bioinformatics 19(12): 1572–1574. 10.1093/bioinformatics/btg180 12912839

[B66] Strzałka B Jankowiak R Bilański P Patel N Hausner G Linnakoski R Solheim H (2020) Two new species of Ophiostomatales (Sordariomycetes) associated with the bark beetle *Dryocoetesalni* from Poland. MycoKeys 68: 23–48. 10.3897/mycokeys.68.50035 32607057 PMC7314864

[B67] Swofford DL (2003) PAUP* 4.0: phylogenetic analysis using parsimony (*and other methods). Sinauer Associates, Sunderland.

[B68] Taerum SJ de Beer ZW Marincowitz S Jankowiak R Wingfield MJ (2018) *Ophiostomaquercus*: An unusually diverse and globally widespread tree-infecting fungus. Fungal Biology 122(9): 900–910. 10.1016/j.funbio.2018.05.005 30115324

[B69] Tedersoo L Bahram M Põlme S Kõljalg U Yorou NS Wijesundera R Ruiz LV Vasco-Palacios AM Thu PQ Suija A Smith ME Sharp C Saluveer E Saitta A Rosas M Riit T Ratkowsky D Pritsch K Põldmaa K Piepenbring M Phosri C Peterson M Parts K Pärtel K Otsing E Nouhra E Njouonkou AL Nilsson RH Morgado LN Mayor J May TW Majuakim L Lodge DJ Lee SS Larsson K-H Kohout P Hosaka K Hiiesalu I Henkel TW Harend H Guo L Greslebin A Grelet G Geml J Gates G Dunstan W Dunk C Drenkhan R Dearnaley J De Kesel A Dang T Chen X Buegger F Brearley FQ Bonito G Anslan S Abell S Abarenkov K (2014) Global diversity and geography of soil fungi. Science 346(6213): e6213. 10.1126/science.1256688 25430773

[B70] Urbanová M Šnajdr J Baldrian P (2015) Composition of fungal and bacterial communities in forest litter and soil is largely determined by dominant trees. Soil Biology & Biochemistry 84: 53–64. 10.1016/j.soilbio.2015.02.011

[B71] Vilgalys R Hester M (1990) Rapid genetic identification and mapping of enzymatically amplified ribosomal DNA from several *Cryptococcus* species. Journal of Bacteriology 172(8): 4238–4246. 10.1128/jb.172.8.4238-4246.1990 2376561 PMC213247

[B72] Wang HM Wang Z Liu F Wu CX Zhang SF Kong XB Decock C Lu Q Zhang Z (2019) Differential patterns of ophiostomatoid fungal communities associated with three sympatric *Tomicus* species infesting pines in south-western China, with a description of four new species. MycoKeys 50: 93–133. 10.3897/mycokeys.50.32653 31043857 PMC6477840

[B73] Weber G Spaaij F Wingfield MJ (1996) *Leptographiumcostaricense* sp. nov., a new species from roots of *Talaumasambuensis* from Costa Rica. Mycological Research 100(6): 732–736. 10.1016/S0953-7562(96)80206-1

[B74] White TJ Bruns T Lee S Taylor J (1990) Amplification and direct sequencing of fungal ribosomal RNA genes for phylogenetics. In: Innis MA Gelfand DH Sninsky JJ White TJ (Eds) PCR Protocols: a Guide to Methods and Applications.Academic Press, San Diego, 315–322. 10.1016/B978-0-12-372180-8.50042-1

[B75] Zhang Y Hagen F Stielow B Rodrigues AM Samerpitak K Zhou X Feng P Yang L Chen M Deng S Li S Liao W Li R Li F Meis JF Guarro J Teixeira M Al-Zahrani HS Pires de Camargo Z Zhang L de Hoog GS (2015) Phylogeography and evolutionary patterns in *Sporothrix* spanning more than 14 000 human and animal case reports. Persoonia 35(1): 1–20. 10.3767/003158515X687416 26823625 PMC4713101

