# Supplementary material for: ﻿Soil-borne Ophiostomatales species (Sordariomycetes, Ascomycota) in beech, oak, pine, and spruce stands in Poland with descriptions of Sporothrixroztoczensis sp. nov., S.silvicola sp. nov., and S.tumida sp. nov
Source: MycoKeys. 2023 May 16;97:41–69. doi: 10.3897/mycokeys.97.97416 (PMC10210257; doi:10.3897/mycokeys.97.97416)
Supplement: Supplementary material 1 — Pinussylvestris branches used to bait Ophiostomatales species from soil in this study [file mycokeys-97-041-s001.docx]

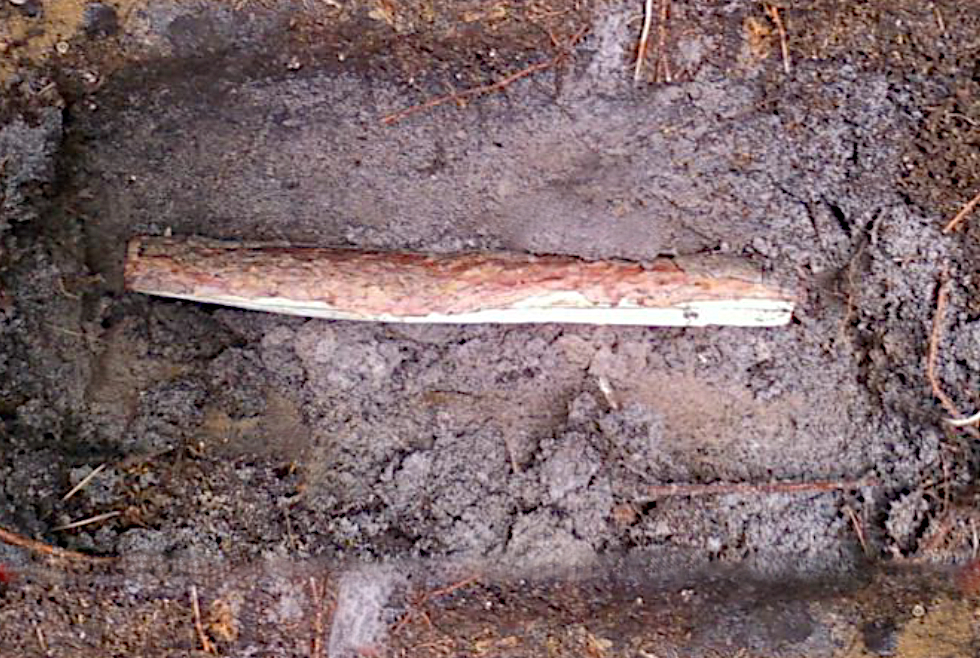


**Figure S1.** An example of a *Pinus sylvestris* branch used to bait Ophiostomatales from soil.


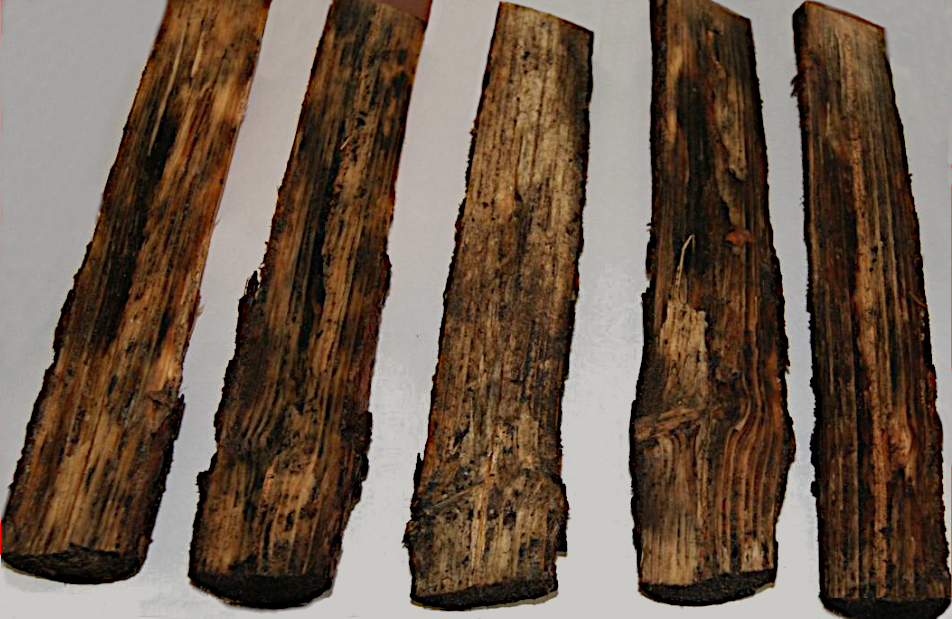


**Figure S2.** *Pinus sylvestris* branches after removal from soil.
