# Supplementary material for: ﻿Soil-borne Ophiostomatales species (Sordariomycetes, Ascomycota) in beech, oak, pine, and spruce stands in Poland with descriptions of Sporothrixroztoczensis sp. nov., S.silvicola sp. nov., and S.tumida sp. nov
Source: MycoKeys. 2023 May 16;97:41–69. doi: 10.3897/mycokeys.97.97416 (PMC10210257; doi:10.3897/mycokeys.97.97416)
Supplement: Supplementary material 2 — Phylograms from Maximum Likelihood analyses [file mycokeys-97-041-s002.docx]

**Figure S3.** Phylogram from Maximum Likelihood (ML) analyses of LSU data for *Leptographium* spp. Sequences obtained in this study are in bold. Bootstrap values ≥ 75% for ML and Maximum Parsimony (MP) analyses are presented at nodes as follows: ML/MP. Bold branches indicate posterior probabilities values ≥ 0.95 obtained from Bayesian Inference (BI) analyses. * Bootstrap values <75%. The tree is drawn to scale (see bar) with branch lengths measured in the number of substitutions per site. *Grosmannia penicillata* and *G. abietina* represent the outgroup.

**Figure S4.** Phylogram from Maximum Likelihood (ML) analyses of ITS data for *Ophiostoma* spp. Sequences obtained in this study are in bold. Bootstrap values ≥ 75% for ML and Maximum Parsimony (MP) analyses are presented at nodes as follows: ML/MP. Bold branches indicate posterior probabilities values ≥ 0.95 obtained from Bayesian Inference (BI) analyses. * Bootstrap values <75%. The tree is drawn to scale (see bar) with branch lengths measured in the number of substitutions per site. *Heinzbutinia* spp. represent the outgroup.

**Figure S5.** Phylogram from Maximum Likelihood (ML) analyses of *TUB*2 data for the *Ophiostoma ulmi* species complex. Sequences obtained in this study are in bold. Bootstrap values ≥ 75% for ML and Maximum Parsimony (MP) analyses are presented at nodes as follows: ML/MP. Bold branches indicate posterior probabilities values ≥ 0.95 obtained from Bayesian Inference (BI) analyses. * Bootstrap values <75%. The tree is drawn to scale (see bar) with branch lengths measured in the number of substitutions per site. *Heinzbutinia* spp. represent the outgroup.

**Figure S6.** Phylogram from Maximum Likelihood (ML) analyses of *TUB*2 data for the *Leptographium procerum* species complex. Sequences obtained in this study are in bold. Bootstrap values ≥ 75% for ML and Maximum Parsimony (MP) analyses are presented at nodes as follows: ML/MP. Bold branches indicate posterior probabilities values ≥ 0.95 obtained from Bayesian Inference (BI) analyses. * Bootstrap values <75%. The tree is drawn to scale (see bar) with branch lengths measured in the number of substitutions per site. *Leptographium serpens* and *L. alacre* represent the outgroup.

**Figure S7.** Phylogram from Maximum Likelihood (ML) analyses of *TEF*1 data for the *Leptographium procerum* species complex. Sequences obtained in this study are in bold. Bootstrap values ≥ 75% for ML and Maximum Parsimony (MP) analyses are presented at nodes as follows: ML/MP. Bold branches indicate posterior probabilities values ≥ 0.95 obtained from Bayesian Inference (BI) analyses. * Bootstrap values <75%. The tree is drawn to scale (see bar) with branch lengths measured in the number of substitutions per site. *Leptographium serpens* and *L. alacre* represent the outgroup.

**Figure S8.** Phylogram from Maximum Likelihood (ML) analyses of *TUB*2 data for the *Leptographium galeiforme* species complex. Sequences obtained in this study are in bold. Bootstrap values ≥ 75% for ML and Maximum Parsimony (MP) analyses are presented at nodes as follows: ML/MP. Bold branches indicate posterior probabilities values ≥ 0.95 obtained from Bayesian Inference (BI) analyses. * Bootstrap values <75%. The tree is drawn to scale (see bar) with branch lengths measured in the number of substitutions per site. *Leptographium olivaceum* and *L. cucullatum* represent the outgroup.

**Figure S9.** Phylogram from Maximum Likelihood (ML) analyses of *TEF*1 data for the *Leptographium galeiforme* species complex. Sequences obtained in this study are in bold. Bootstrap values ≥ 75% for ML and Maximum Parsimony (MP) analyses are presented at nodes as follows: ML/MP. Bold branches indicate posterior probabilities values ≥ 0.95 obtained from Bayesian Inference (BI) analyses. * Bootstrap values <75%. The tree is drawn to scale (see bar) with branch lengths measured in the number of substitutions per site. *Leptographium olivaceum* and *L. cucullatum* represent the outgroup.
